# Exercise prescriptions for patients on hemodialysis in Brazil: a scoping review

**DOI:** 10.1590/2175-8239-JBN-2024-0049en

**Published:** 2024-09-20

**Authors:** Heitor S. Ribeiro, Francini P. Andrade, Diogo V. Leal, Juliana S. Oliveira, Kenneth R. Wilund, Maycon M. Reboredo, João L. Viana

**Affiliations:** 1 University of Maia, Research Center in Sports Sciences, Health Sciences and Human Development, CIDESD, Maia, Portugal. University of Maia, Research Center in Sports Sciences Health Sciences and Human Development, CIDESD Maia Portugal; 2 University of Brasília, Faculty of Medicine, Brasília, DF, Brazil. University of Brasília Faculty of Medicine Brasília DF Brazil; 3 Universidade Federal do Rio Grande do Sul, Ciências Pneumológicas Post-Graduation Program, Porto Alegre, RS, Brazil. Universidade Federal do Rio Grande do Sul Ciências Pneumológicas Post-Graduation Program Porto Alegre RS Brazil; 4 The University of Sydney and Sydney Local Health District, Institute for Musculoskeletal Health, Sydney, Australia. The University of Sydney and Sydney Local Health District Institute for Musculoskeletal Health Sydney Australia; 5 University of Illinois Urbana-Champaign, Department of Kinesiology and Community Health, Urbana, USA. University of Illinois Urbana-Champaign Department of Kinesiology and Community Health Urbana USA; 6 Federal University of Juiz de Fora, School of Medicine, Juiz de Fora, MG, Brazil. Federal University of Juiz de Fora School of Medicine Juiz de Fora MG Brazil

**Keywords:** Renal Insufficiency, Chronic, Dialysis, Exercise, Resistance Training, Endurance Training

## Abstract

**Introduction:**

Exercise is being incorporated into the treatment of patients on hemodialysis; however, little is known about the major characteristics of these interventions.

**Objective:**

To describe the exercise protocols prescribed for hemodialysis patients in Brazil.

**Methods:**

A scoping review was conducted following JBI and Prisma-ScR guidelines. Searches were carried out in Medline, Embase and three other databases until May 2024. Other sources (websites, books and guidelines) were also investigated. Evidence from patients on hemodialysis describing exercise protocols in all settings and designs in Brazil was included.

**Results:**

Forty-five pieces of evidence were found, resulting in 54 exercise protocols from 16 Brazilian states. Strength exercises (33.3%), followed by aerobic exercises (22.2%), were the most prescribed, mainly to be performed during dialysis (85.2%). The most prevalent professionals supervising the programs were physiotherapists and exercise physiologists (37.0% and 18.5%, respectively). All protocols implemented the principles of type and frequency training, while progression was adopted in only 53.7%. The main prescription was three times per week (88.9%). Exercise intensity was predominantly determined by subjective methods (33.3%).

**Conclusion:**

Aerobic and strength exercises during dialysis were the most commonly prescribed modalities in Brazil, with the majority of programs being properly supervised by qualified professionals. However, existing protocols have not employed systematic progression throughout the intervention, which would be appropriate for providing better physiological responses and adaptations.

## Introduction

Hemodialysis is the most commonly prescribed kidney replacement therapy in Brazil. Brazilian data from the 2022 dialysis survey revealed that 95% of patients with kidney failure were undergoing hemodialysis^1^. Patients on hemodialysis commonly experience sedentary behavior^2^ and physical disability^3,4,5,6^, which increases their morbidity and mortality. Exercise interventions have been introduced as an attempt to change this scenario^7^, but their implementation as part of the treatment routine is still lacking^8^. Furthermore, there is a need for evidence that describes exercise protocols in detail to support the expansion of viable programs.

Previously, results of a worldwide analysis from a scoping review showed that Brazil was the leading country in reporting exercise protocols for this population^9^. However, Barros et al.^10^ recently found that only 16% of dialysis centers in Brazil had an intradialytic exercise program integrated into their clinical routine. In an effort to increase awareness and knowledge within the Brazilian Society of Nephrology to support the implementation of exercise programs, we conducted a secondary analysis of a scoping review to describe exercise prescriptions for patients on hemodialysis in Brazil.

## Methods

The protocol for our scoping review and the article with global data have been previously published^9,11^, and further details on the methodology can be found in those publications. Briefly, the JBI^12^ and Prisma-ScR^13^ guidelines for scoping reviews were adopted. A comprehensive search strategy was conducted in the Medline, Embase, SportDiscus, Cinahl and Lilacs databases using terms related to “hemodialysis”, “dialysis”, “physical exercise” and “physical training”. Full search strategy for each database may be seen in Supplementary Material 1 The databases were searched from inception until December 2021, and additional manual searching for new Brazilian evidence was undertaken until May 2024.

The PCC framework was followed, selecting any evidence from reports/studies with adults on hemodialysis (participants), prescribing exercise interventions (concept), in all settings and study designs in Brazil (context). Two independent reviewers (HR and FA) selected titles and abstracts. Full-text reading was performed by the primary reviewer (HR). Discrepancies were discussed with an additional reviewer (DL).

Relevant data were extracted by the lead reviewer (HR), and double-checked by others (FA and DL) using an adapted spreadsheet^14^. Those data included details on exercise prescription (e.g. type, frequency, duration, location, volume, progression, intensity, professionals involved, periodization, etc.). Discrepancies were resolved with an additional reviewer (JV).

The data were described according to Prisma-ScR guidelines^13^. Protocols were analyzed according to the exercise principles outlined by the American College of Sports Medicine (ACSM) (FITT-VP: frequency, intensity, time, type, volume, and progression)^15^. A more in-depth description of the data analysis can be found in another publication^9^.

## Results

### Selection of Studies

**Figure 1:**
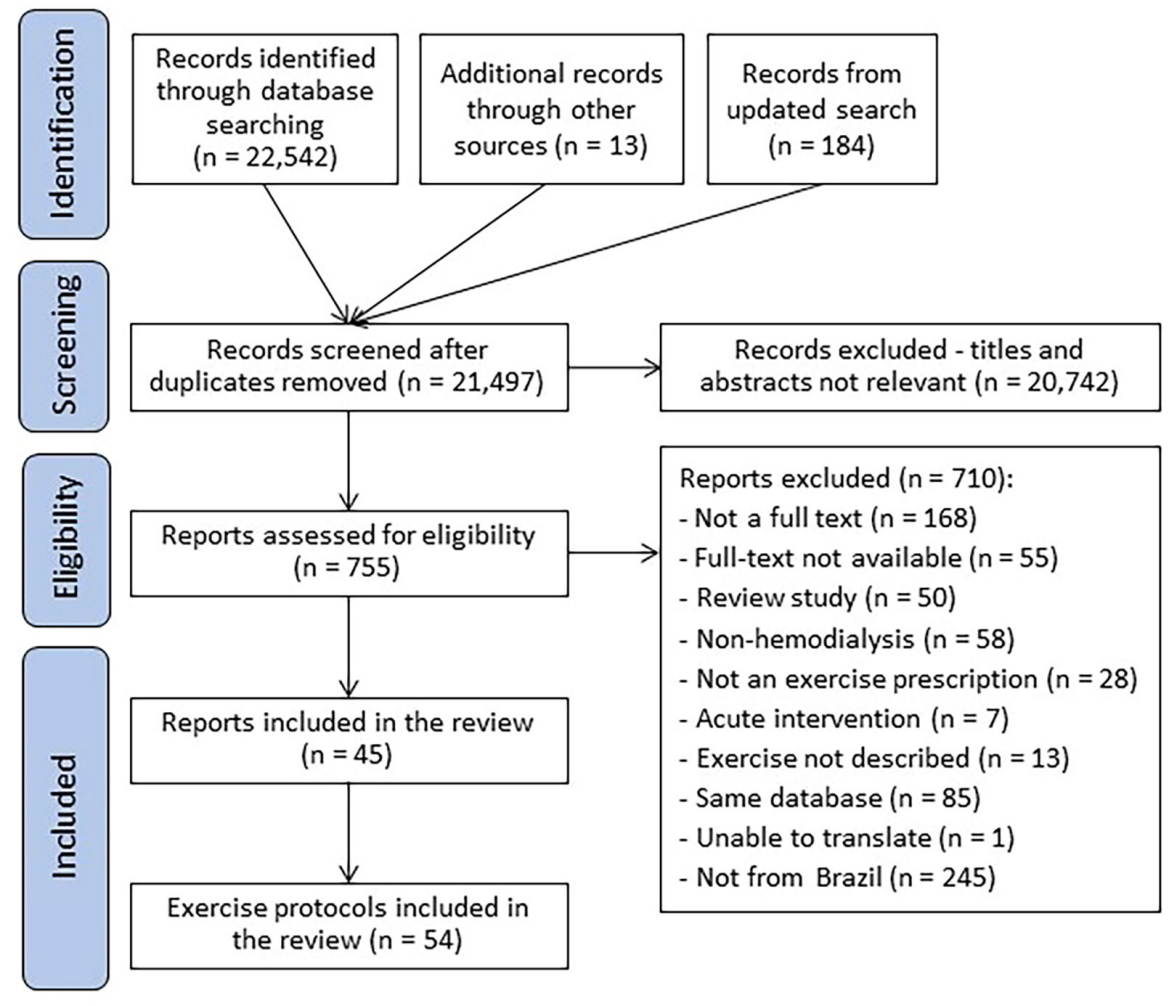
Flowchart of the scoping review.

A total of 21,312 records were found; of these, 285 were included in our main global review^8^. From Brazil, 39 were included in this secondary analysis. In an additional search conducted in May 2024, 6 articles published between 2022 and 2024 were identified, yielding 54 exercise protocols (Figure 1; complete list of articles in Supplementary Material 2). All exercise protocols were reported in original articles.

### Characteristics

**Figure 2:**
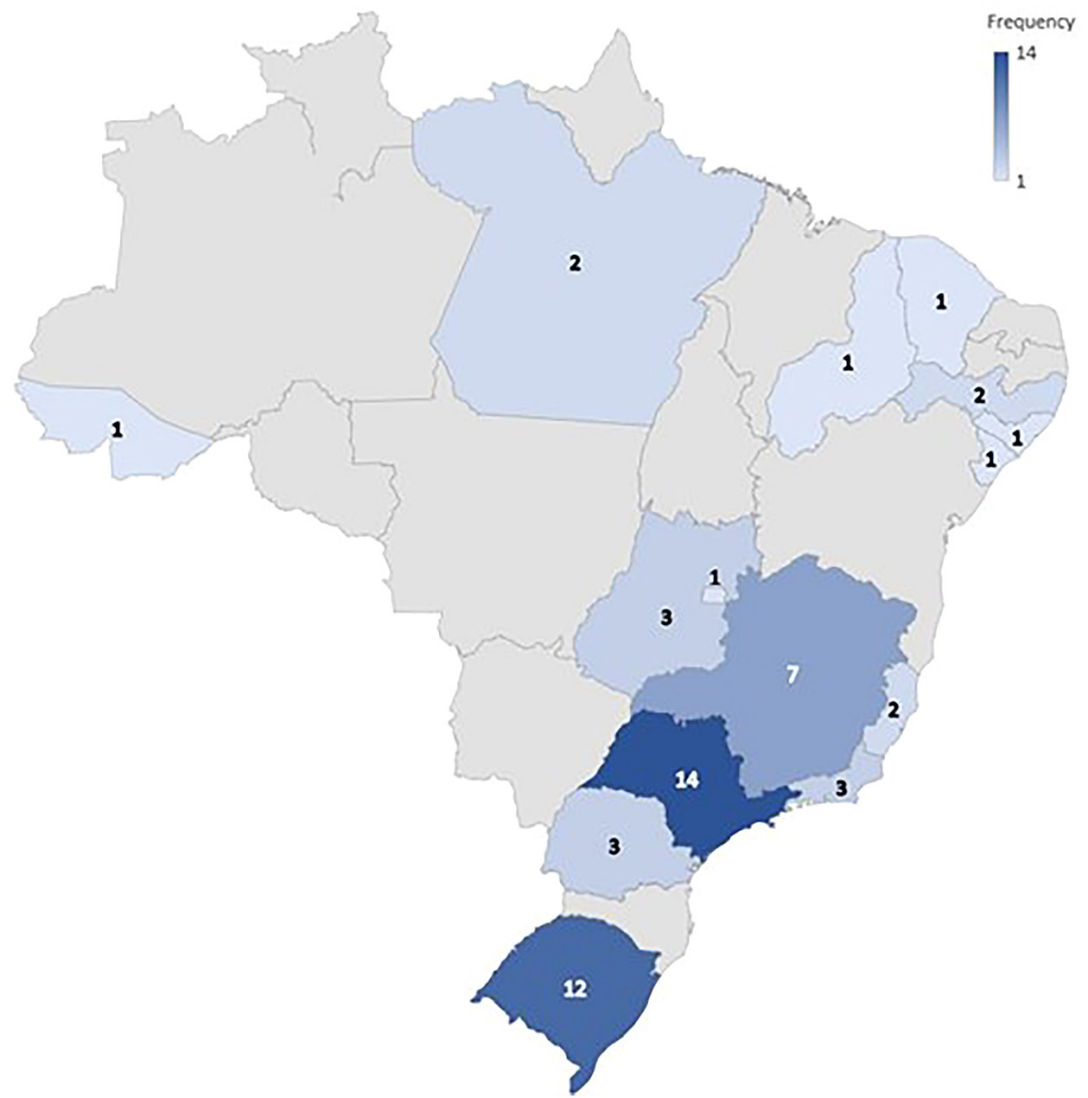
Geographical heat map of exercise protocols prescribed for patients on hemodialysis in Brazil.

The included reports originated from 16 states and all regions of Brazil. Figure 2 shows that the most prevalent state was São Paulo (n = 14; 25.9%). Strength training (n = 18; 33.3%) was the most frequently prescribed type. Table S2 shows the characteristics of the included reports.

### General Findings

Table 1 outlines the key characteristics of the exercise programs. The median duration of interventions was 12 [interquartile range: 8 – 13] weeks. Exercise was predominantly prescribed during dialysis (i.e. intradialytic; n = 45; 83.3%). For exercise programs that prescribed any intradialytic component, the intervention was mainly performed during the first half of the dialysis session (n = 35; 77.8%). The most prevalent professionals supervising exercise programs were physiotherapists and exercise physiologists (n = 20; 37.0%, and n = 10; 18.5%, respectively).

**Table 1:** Characteristics of the exercise protocols

	Aerobic (n = 12)	Strength (n = 18)	Combined* (n = 6)		Mobility (n = 3)	Respiratory (n = 8)	Neuromuscular electrical stimulation (n = 5)	Virtual reality (n = 1)	Vibration platform (n = 1)
Duration (weeks), median [IQR]	12 [12 – 17]	12 [10 – 26]	12 [8 – 44]		13 [8 – 13]	8 [8 – 12]	8	12	12
**Setting**, n (%)
Intradialytic^#^	12 (100)	15 (83.3)	5 (83.3)		2 (66.7)	6 (75.0)	5 (100)	1 (100)	0
Interdialytic	0	0	1 (16.7)		0	1 (12.5)	0	0	1 (100)
Pre-hemodialysis	0	3 (16.7)	0		0	0	0	0	0
Post hemodialysis	0	0	0		0	0	0	0	0
Pre- and intradialytic	0	0	0		0	0	0	0	0
Home	0	0	0		0	0	0	0	0
Home and intradialytic	0	0	0		0	1 (12.5)			
Non-reported	0	0	0		1 (33.3)	0	0	0	0
**Moment of dialysis**^†^, n (%)									
First half	10 (83.3)	11 (73.3)	4 (80.0)		2 (100)	5 (62.5)	4 (80.0)	1 (100)	–
Second half	0	3 (20.0)	0		0	0	0	0	–
Non-reported	2 (16.7)	2 (13.3)	1 (20)		0	1 (12.5)	1 (20.0)	0	–
**Professional supervision**^†^, n (%)									
Physiotherapist	0	5 (27.8)	5 (83.3)		3 (100)	3 (37.5)	3 (60.0)	1 (100)	0
Exercise physiologist	3 (25.0)	6 (33.3)	1 (16.7)		0	0	0	0	0
Healthcare professional	2 (16.7)	4 (22.2)	0		0	0	0	0	0
Non-supervised	0	0	0		0	1 (12.5)	0	0	0
Non-reported	8 (66.7)	8 (44.4)	0		0	5 (62.5)	2 (40.0)	0	1 (100)
**Components of the session**, n (%)			**Aerobic**	**Strength**					
Warm-up	6 (50.0)	5 (27.8)	4 (66.7)	1 (16.7)	0	0	4 (80.0)	0	0
Conditioning	11 (91.7)	14 (77.8)	6 (100)	5 (83.3)	2 (66.7)	8 (100)	5 (100)	1 (100)	1 (100)
Cool down	6 (50.0)	3 (16.7)	2 (33.3)	1 (16.7)	0	0	3 (60.0)	0	0

Abbreviation: IQR, interquartile range.

Notes: *Combining aerobic and strength training.

^#^Only during dialysis, not including other settings.

^†^The sum may exceed 100% because some exercise programs included more than one type.

There are missing values due to the absence of data in some included studies.

**Figure 3:**
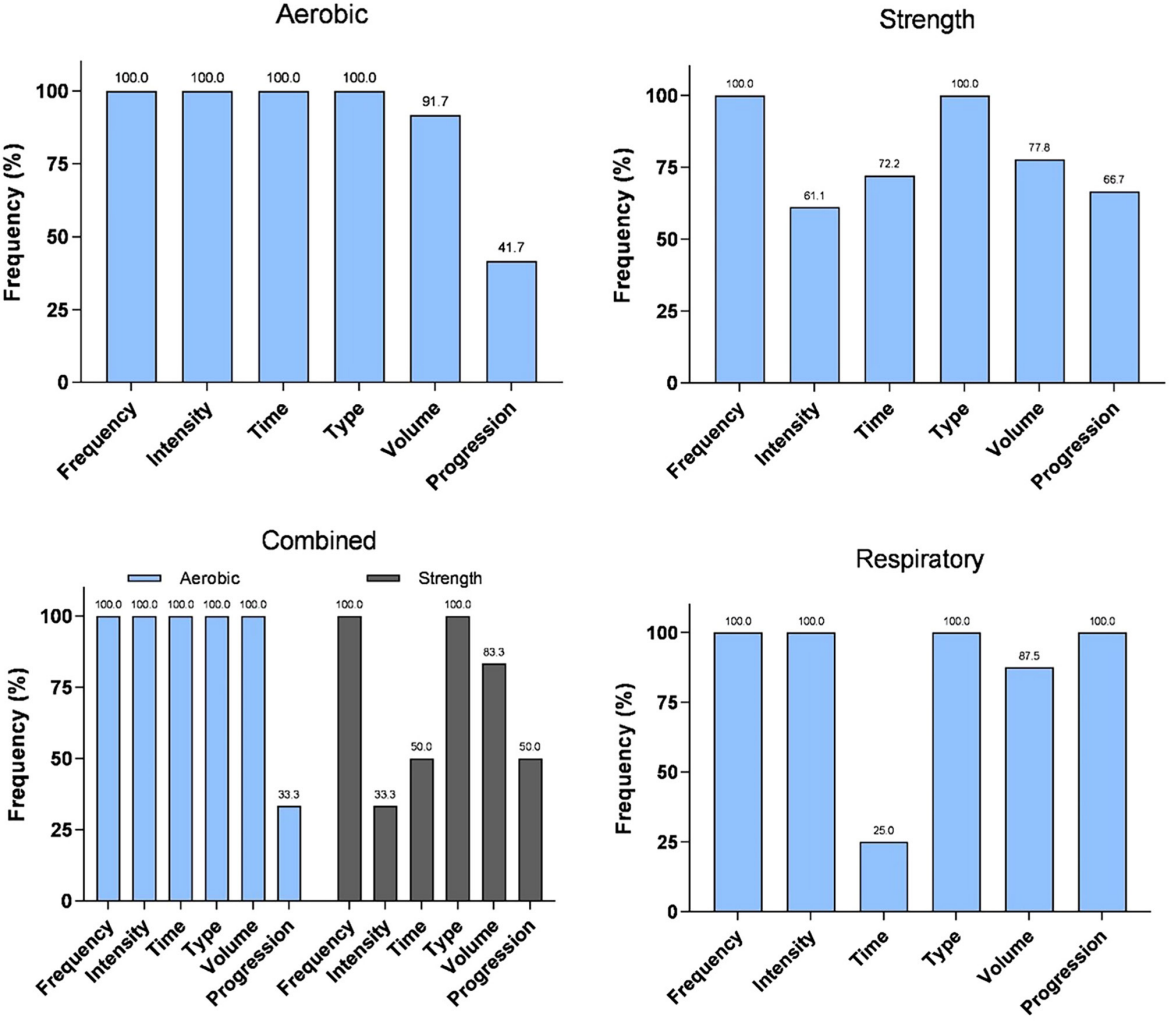
FITT-VP training principles (ACSM) adopted in the most prescribed protocols.

Abbreviations: ACSM, American College of Sports Medicine; FITT-VP, Frequency, Intensity, Time, Type, Volume, and Progression.

The principles of exercise adopted in the prescribed protocols are shown in Figure 3. All protocols included type and frequency, but only 53.7% (n = 29) considered progression. Most protocols prescribed exercises three days a week (n = 47; 88.9%) and used subjective methods (n = 18; 33.3%) to determine exercise intensity, while 14 (25.9%) protocols did not report intensity control.

### Characteristics of Exercise Protocols

#### Aerobic

The cycle ergometer was used in all aerobic protocols (n = 12; 100%), and one included partial restriction of blood flow in the lower limbs. All protocols prescribed exercises three days per week, and most of them monitored exercise intensity by objective methods (n = 9; 75.0%), primarily through heart rate scales (n = 9; 75.0%), and rate of perceived exertion (RPE) (n = 6; 50.0%).

#### Strength

Six strength protocols included lower limb exercises (33.3%), and 11 combined exercises for both lower and upper limbs (61.1%). Six protocols used dumbbells and free weights (33.3%). Two studies incorporated bodyweight exercises (11.1%). Most protocols prescribed exercises three days a week (n = 16; 88.9%), and monitored exercise intensity using subjective methods through RPE scales (n = 10; 55.6%).

#### Combined

All combined protocols prescribed both aerobic and strength training within the same session (n = 6; 100%), with aerobic training being predominantly performed first (n = 5; 83.3%).

#### Aerobic Component

Cycling was prescribed for all protocols, and one also included walking/running on a treadmill^16^. Five protocols prescribed exercises three days a week (83.3%), and most of them monitored exercise intensity using subjective methods (n = 5; 83.3%), mainly through RPE scales (n = 4; 66.7%).

#### Strength Component

Four protocols included exercises for both lower and upper limbs (66.6%), while two focused only on the lower limbs (33.3%). Most protocols used dumbbells and free weights (n = 5; 83.3%), and one incorporated elastic bands or balls (16.7%). Five protocols prescribed exercises three days a week (83.3%), and only three described exercise intensity monitoring (50%).

#### Respiratory

Respiratory interventions (n = 8) were primarily performed three days a week (n = 6; 75.0%) with objective intensity methods applied in all of them, mainly through maximal inspiratory pressure (n = 7; 87.5%), ranging from 40 to 70%.

#### Mobility

Mobility protocols (n = 3) were mainly prescribed three days per week (n = 2; 66.3%), with duration ranging from 25 to 45 minutes. No intensity or progression approach was described.

#### Neuromuscular Electrical Stimulation

Five protocols employed neuromuscular electrical stimulation three days a week (100%), with sessions lasting between 20 and 60 minutes each. The intensity was objectively prescribed through pulse rate (100%), which ranged from 20 to 80 Hz.

#### Virtual Reality

A single protocol used non-immersive exergames and was prescribed three days per week. The exercise intensity was monitored through RPE, ranging from 12 to 14 on the Borg scale of 6 to 20, representing a somewhat hard to hard intensity.

#### Vibration Platform

One exercise protocol used vibration platforms at a frequency of 35 Hz. The protocol consisted of performing consecutive 30-second isometric semi-squats separated by 30-second rest periods for a total of 10 to 20 minutes, twice a week.

## Discussion

Our scoping review described the exercise protocols prescribed for patients on hemodialysis in Brazil. Evidence came mainly from the states of São Paulo and Rio Grande do Sul, with strength training being the most commonly prescribed type of exercise. Physiotherapists supervised the majority of protocols. The principle of systematic progression was applied in only half of protocols, and exercise intensity was predominantly determined by subjective methods. The exercise frequency was primarily three times a week and during dialysis, highlighting the need for a more holistic approach to exercise and physical activity in this population, including a lifestyle that involves “moving more”^17^.

Unlike the global analyses, strength training, rather than aerobic exercises, was the most prescribed type among the included protocols^9^. Aerobic exercise often requires minimal professional supervision and incurs lower costs. In Brazil, exercise can only be prescribed and supervised by physiotherapists and/or exercise physiologists. This suggests that strength exercises have been more widely adopted than in other countries across the world. Additionally, they are more affordable and require simpler equipment when compared to aerobic exercises using a cycle ergometer. However, despite such professional support, other types of exercise programs (e.g. neuromuscular electrical stimulation, virtual reality, and vibration platforms) have been poorly prescribed. Such interventions are generally expensive and require modern equipment, making their implementation in clinical practice more challenging.

In our global analysis^9^, a large number of protocols did not properly describe the exercise-related variables recommended by the ACSM (i.e. FITT-VP principles)^15^. However, in this Brazilian analysis, there was a greater number of exercise protocols that followed all or most of those principles. Nevertheless, progression was little adopted in both aerobic and combined exercise programs. Proper exercise progression plays a major role in producing long-term physiological adaptations and may have an impact on health benefits^18,19^. Therefore, efforts from Brazilian societies of nephrology, physiotherapy, and exercise physiology are needed to provide guidelines on the implementation, prescription, monitoring, and supervision of exercises for patients undergoing hemodialysis, as recently done by the UK Renal Association^20^. The newly created *Grupo Brasileiro de Reabilitação em Nefrologia* (GBREN, Brazilian Group for Rehabilitation in Nephrology)^21,22^ has so far operated as a collaborative network to support exercise professionals working in this area. In clinical practice, until there is a national guideline, we recommend that professionals involved in the prescription and supervision of physical exercise for this population follow recommendations such as those of the ACSM^15^.

To the best of our knowledge, this is the first scoping review describing the evidence on how exercise has been prescribed for patients on hemodialysis in Brazil. We followed well-known guidelines, conducted a comprehensive search strategy, and included most types of exercise, which contributed to a robust number of studies included. Despite these strengths, there are some limitations. The full text was read only by the lead reviewer; however, to minimize this bias, the data extraction was double-checked by two other reviewers. In addition, the search strategy did not include any Brazilian-specific terms or the Brazilian Portuguese language, which may have impacted the capture of non-English publications.

In conclusion, strength and aerobic exercise interventions were the most commonly prescribed in Brazil, although other modalities have recently been prescribed. However, we found a low number of protocols adopting systematic progression of exercise training principles over the course of the intervention. Future protocols should adopt these criteria to ensure better physiological responses and adaptations. Therefore, we believe our findings could provide the nephrology community with a better understanding of how to provide, implement, monitor, and supervise exercise programs for individuals undergoing hemodialysis. Furthermore, public policies should encourage the increased presence of qualified professionals to supervise exercise programs (namely, physiotherapists and exercise physiologists).

## Supplementary Material

The following online material is available for this article:

Supplementary Material 1 – Search strategies.

Supplementary Material 2 – References of the included reports.

Table S1 – Preferred Reporting Items for Systematic reviews and Meta-Analyses extension for Scoping Reviews (Prisma-ScR) Checklist.

Table S2 – Characteristics of the exercise protocols.
