# Supplementary material for: Exercise prescriptions for patients on hemodialysis in Brazil: a scoping review
Source: J Bras Nefrol. 2024 Sep 20;46(4):e20240049. doi: 10.1590/2175-8239-JBN-2024-0049en (PMC11420935; doi:10.1590/2175-8239-JBN-2024-0049en)
Supplement: Supplementary file 1 [file 2175-8239-jbn-46-4-e20240049-supp01.pdf]

Braz. J. Nephrol. (J. Bras. Nefrol.)
https://doi.org/10.1590/2175-8239-JBN-2024-0049en

Supplementary Material to "Exercise prescriptions for patients on hemodialysis in Brazil: a scoping review"

Supplementary Material 1. Search strategies.

MEDLINE – 5,369 references (Dated 17 December 2021)

1. exp renal insufficiency, chronic/ or exp kidney diseases/
2. end-stage renal or end-stage kidney or endstage renal or endstage kidney or chronic kidney or chronic renal
3. ESRF or ESKF or ESRD or ESKD or CKF or CKD or CRF or CRD
4. exp renal replacement therapy/ or exp renal dialysis
5. dialysis or h?emodialysis or h?emofiltration or h?emodiafiltration
6. or/1-5
7. exp exercise/ or exp exercise therapy
8. (exercise or resistance training or strength training or aerobic training or endurance training or cycling training or combined exercise training or physical rehabilitation or physiotherapy or physical therapy).mp.
9. or/7-8
10. 6 and 9
11. limit 10 to humans

Embase (OvidSP) – 15,988 references (Dated 20 December 2021)

1. exp renal insufficiency, chronic/ or exp kidney diseases/
2. ((kidney or renal) adj5 (disease* or injur* or insufficienc* or failure*)).mp
3. (end-stage renal or end-stage kidney or endstage renal or endstage kidney or chronic kidney or chronic renal).mp
4. (ESRF or ESKF or ESRD or ESKD or CKF or CKD or CRF or CRD).mp
5. exp renal replacement therapy/ or exp renal dialysis
6. (dialysis or h?emodialysis or h?emofiltration or h?emodiafiltration).mp
7. or/1-6
8. exp exercise/ or exp exercise therapy
9. (exercise or resistance training or strength training or aerobic training or endurance training or cycling training or combined exercise training or physical rehabilitation or physiotherapy or physical therapy).mp.
10. or/8-9
11. 7 and 10
12. limit 11 to human

CINAHL (EBSCO) – 972 references (Dated 20 December 2021)

1. (MH "Renal Insufficiency+")
2. MH renal insufficienc* OR kidney insufficienc* OR renal diseas* OR kidney diseas*
3. MH chronic kidney disease OR chronic renal insufficiency
4. MH CKF OR CKD OR CRF OR CRD

5. S1 OR S2 OR S3 OR S4
6. MH exercise OR exercise physical
7. MH resistance training OR strength training OR aerobic training OR cycling training OR combined exercise training OR physical rehabilitation OR physiotherapy OR physical therapy
8. S6 OR S7
9. S5 AND S8

LILACS (BVS) – 11 references (Dated 20 December 2021)

(Renal Insufficiency OR Renal Insufficiency, Chronic OR Kidney Failure, Chronic OR renal replacement therapy OR Continuous Renal Replacement Therapy OR Hemofiltration OR Hemoperfusion OR Hybrid Renal Replacement Therapy OR Intermittent Renal Replacement Therapy OR Renal Dialysis) AND (exercise OR exercise physical OR resistance training OR strength training OR aerobic training OR cycling training OR combined exercise training OR physical rehabilitation OR physiotherapy OR physical therapy)

SPORTDiscuss (EBSCO) – 202 references (Dated 20 December 2021)

1. Hemodialysis
2. TI hemodialysis OR AB hemodialysis OR TI haemodialysis OR AB haemodialysis
3. TI renal insufficiency OR AB renal insufficiency
4. TI kidney failure OR AB kidney failure OR TI renal failure OR AB renal failure
5. TI renal replacement therapy OR AB renal replacement therapy OR TI kidney replacement therapy OR AB kidney replacement therapy
6. 1-5 OR
7. Exercise
8. TI exercise OR AB exercise
9. TI "Physical conditioning" OR AB "Physical conditioning"
10. TI "Resistance training" OR AB "Resistance training"
11. TI "strength training" OR AB "strength training"
12. TI "Functional training" OR AB "Functional training"
13. TI "Aerobic activities" OR AB "Aerobic activities" OR TI "Aerobic activity" OR AB "Aerobic activity"
14. TI "Cardiovascular activities" OR AB "Cardiovascular activities" OR TI "Cardiovascular activity" OR AB "Cardiovascular activity"
15. TI "Endurance activities" OR AB "Endurance activities" OR TI "Endurance activity" OR AB "Endurance activity"
16. 7-15 OR
17. S6 AND S16
