# Supplementary material for: Exercise prescriptions for patients on hemodialysis in Brazil: a scoping review
Source: J Bras Nefrol. 2024 Sep 20;46(4):e20240049. doi: 10.1590/2175-8239-JBN-2024-0049en (PMC11420935; doi:10.1590/2175-8239-JBN-2024-0049en)
Supplement: Supplementary file 2 [file 2175-8239-jbn-46-4-e20240049-supp02.pdf]

Braz. J. Nephrol. (J. Bras. Nefrol.)
https://doi.org/10.1590/2175-8239-JBN-2024-0049en

Supplementary Material to "Exercise prescriptions for patients on hemodialysis in Brazil: a scoping review"

Supplementary Material 2. References of the included reports.

1. Reboredo M M, Pinheiro BV, Neder JA, Ávila MP, Araujo e Ribeiro ML, Mendonça AF, et al. Effects of aerobic training during hemodialysis on heart rate variability and left ventricular function in end-stage renal disease patients. Brazilian J Nephrol. 2010;32(4):367-73. PubMed PMID: 21541451.

2. Rocha ERE, Magalhães SM, de Lima VP. Repercussion of physiotherapy intradialytic protocol for respiratory muscle function, grip strength and quality of life of patients with chronic renal diseases. Brazilian J Nephrol. 2010;32(4):355-66. PubMed PMID: 21541450.

3. Giendruczak V, Regina J. Effects of inspiratory muscle training in hemodialysis patients. Brazilian J Nephrol. 2010;33(1):45-51.

4. Martins CTB, Ramos GSM, Guaraldo SA, Uezima CBB, Martins JPLB, Ribeiro Jr E. Comparison of cognitive function between patients on chronic hemodialysis who carry out assisted physical activity and inactive ones. Brazilian J Nephrol Nephrol. 2011;33(1):27-30. doi: http://doi.org/10.1590/S0101-28002011000100003. PubMed PMID: 21541459.

5. Padulla SAT, Matta MV, Melatto T, Miranda RCV, Camargo MR. A fisioterapia pode influenciar na qualidade de vida de indivíduos em hemodiálise? Ciência. Cuid e Saúde. 2011;10(3):564-70.

6. Soares K, Viesser M, Rzniski T, Brum E. Efficacy of a physical exercises protocol in patients with chronic renal failure during treatment of hemodialysis, valued by SF-36. Fisioter Mov. 2011;24(1):133-40. doi: http://doi.org/10.1590/S0103-51502011000100015.

7. Marchesan M, Rombaldi AJ. Programa De Exercícios Físicos Para O Doente Renal Crônico Em Hemodiálise. Rev Bras Atividade Física Saúde. 2012;17(1):75. doi: http://doi.org/10.12820/rbafs.v.17n1p75-78.

8. Orcy RB, Dias PS, Seus TL, Barcellos FC, Bohlke M. Combined resistance and aerobic exercise is better than resistance training alone to improve functional performance of haemodialysis patients - results of a randomized controlled trial. Physiother Res Int. 2012;17(4):235-43. doi: http://doi.org/10.1002/pri.1526. PubMed PMID: 22693148.

9. da Silva SF, Pereira AA, da Silva WAH, Simôes R, Barros No JR. Physical therapy during hemodialysis in patients with chronic kidney disease. J Bras Nefrol. 2013;35(3):170-6. doi: http://doi.org/10.5935/0101-2800.20130028. PubMed PMID: 24100735.

10. Moraes C, Leal VO, Marinho SM, Barroso SG, Rocha GS, Boaventura GT, et al. Resistance exercise training does not affect plasma irisin levels of hemodialysis patients. Horm Metab Res. 2013;45(12):900-4. doi: http://doi.org/10.1055/s-0033-1354402. PubMed PMID: 24013946.

11. Lima FF, Miranda RCV, Rossi E, Silva RC, Monteiro HL, Yen LS, et al. Functional evaluation pre and post physical exercise program for patients in hemodialysis. Med. 2013;46(1):24-35.

12. Ribeiro R, Coutinho GL, Iuras A, Barbosa AM, de Souza JAC, Diniz DP, et al. Effect of resistance exercise intradialytic in renal patients chronic in hemodialysis. J Bras Nefrol. 2013;35(1):13-9. doi: http://doi.org/10.5935/01012800.20130003. PubMed PMID: 23598747.

13. Pellizzaro CO, Thomé FS, Veronese FV. Effect of peripheral and respiratory muscle training on the functional capacity of hemodialysis patients. Ren Fail. 2013;35(2):189-97. doi: http://doi.org/10.3109/0886022X.2012.745727. PubMed PMID: 23199095.

14. De Lima MC, De Lima Cicotoste C, Da Silva Cardoso K, Forgiarini Jr LA, Monteiro MB, Dias AS. Effect of exercise performed during hemodialysis: strength versus aerobic. Ren Fail. 2013;35(5):697-704. doi: http://doi.org/10.3109/0886022X.2013.780977. PubMed PMID: 23560491.

15. Tomich GM, Bernardino LS, Ferreira FO. Impact of physical therapy on functional capacity and life quality of patients with chronic kidney disease. Fisioter Mov. 2014;27(4):643-51. doi: http://doi.org/10.1590/0103-5150.027.004.AO16.

16. Ferreira R, Ribeiro M, Costa T, Silva C. Adaptações crônicas de um protocolo de treino aeróbio durante a hemodiálise. Rev Inspirar Mov Saúde. 2015;7(3):25-30.

17. Roxo RS, Xavier VB, Miorin LA, Magalhães AO, Sens YADS, Alves VLDS. Impact of neuromuscular electrical stimulation on functional capacity of patients with chronic kidney disease on hemodialysis. J Bras Nefrol. 2016;38(3):344-50. doi: http://doi.org/10.5935/0101-2800.20160052. PubMed PMID: 27737393.

18. Guio BM, Gomes CP, da Costa FB, de Oliveira ADS, Duarte MT, Leite Júnior M. Benefcial effects of intradialytic cardiopulmonary rehabilitation. J Bras Nefrol. 2017;39(3):275-82. doi: http://doi.org/10.5935/0101-2800.20170051. PubMed PMID: 29044337.

19. Campos NG, Marizeiro DF, Florêncio ACL, Silva ÍC, Meneses GC, Bezerra GF, et al. Effects of respiratory muscle training on endothelium and oxidative stress biomarkers in hemodialysis patients: A randomized clinical trial. Respir Med. 2017;2018(134):103-9. doi: http://doi.org/10.1016/j.rmed.2017.12.005. PubMed PMID: 29413495.

20. Sanchez HM, Nascimento DMB, Castro K, Sanchez EGM. Melo Jr JP , Agostinho PLS. Benefits of intradialytic physiotherapy in quality of life, pain, edema and respiratory function of patients with chronic kidney disease. Fisioter Mov. 2018;31(0):3107. doi: http://doi.org/10.1590/1980-5918.031.ao07.

21. Belik F, Oliveirae Silva VR, Braga GP, Bazan R, Perez Vogt B, Costa Teixeira Caramori J, et al. Influence of intradialytic aerobic training in cerebral blood flow and cognitive function in patients with chronic kidney disease: a pilot randomized controlled trial. Nephron. 2018;140(1):9-17. doi: http://doi.org/10.1159/000490005. PubMed PMID: 29879707.

22. Figueiredo PHS, Lima MMO, Costa HS, Martins JB, Flecha OD, Gonçalves PF, et al. Effects of the inspiratory muscle training and aerobic training on respiratory and functional parameters, inflammatory biomarkers, redox status and quality of life in hemodialysis patients: a randomized clinical trial. PLoS One. 2018;13(7):e0200727. doi: http://doi.org/10.1371/journal.pone.0200727. PubMed PMID: 30048473.

23. Rosa CSC, Nishimoto DY, Souza GDE, Ramirez AP, Carletti CO, Daibem CGL, et al. Effect of continuous progressive resistance training during hemodialysis on body composition, physical function and quality of life in end-stage renal disease patients: a randomized controlled trial. Clin Rehabil. 2018;32(7):899-908. doi: http://doi.org/10.1177/0269215518760696. PubMed PMID: 29504416.

24. Morais MJD, Raimundo RD, Oliveira FS, De Abreu LC, Bezerra IMP, Silva RPM, et al. Evaluation of the effects of aerobic training during hemodialysis on autonomic heart rate modulation in patients with chronic renal disease. Medicine (Baltimore). 2019;98(23):1-7. doi: http://doi.org/10.1097/MD.0000000000015976. PubMed PMID: 31169731.

25. Maynard LG, de Menezes DL, Lião NS, de Jesus EM, Andrade NLS, Santos JCD, et al. Effects of exercise training combined with virtual reality in functionality and health-related quality of life of patients on hemodialysis. Games Health J. 2019;8(5):339-48. doi: http://doi.org/10.1089/g4h.2018.0066. PubMed PMID: 31539293.

26. Oliveira e Silva VR, Stringuetta Belik F, Hueb JC, de Souza Gonçalves R, Costa Teixeira Caramori J, Perez Vogt B, et al. Aerobic exercise training and nontraditional cardiovascular risk factors in hemodialysis patients: results from a prospective randomized trial. Cardiorenal Med. 2019;9(6):391-9. doi: http://doi.org/10.1159/000501589. PMid:31597151.

27. Fuzari HK, Dornelas de Andrade A, A Rodrigues M, I Medeiros A, F Pessoa M, Lima AM, et al. Whole body vibration improves maximum voluntary isometric contraction of knee extensors in patients with chronic kidney disease: A randomized controlled trial. Physiother Theory Pract. 2019;35(5):409-18. doi: http://doi.org/10.1080/09593985.2018.1443537. PubMed PMID: 29482412.

28. de Medeiros AIC, Brandão DC, de Souza RJP, Fuzari HKB, Barros CESR, Barbosa JBN, et al. Effects of daily inspiratory muscle training on respiratory muscle strength and chest wall regional volumes in haemodialysis patients: a randomised clinical trial. Disabil Rehabil. 2019;41(26):3173-80. doi: http://doi.org/10.1080/09638288.2018.1485181. PubMed PMID: 30052475.

29. de Castro APA, Barbosa SR, Mansur HN, Ezequiel DGA, Costa MB, de Paula RB. Intradialytic resistance training: an effective and easy-to-execute strategy. J Bras Nefrol. 2019;41(2):215-23. doi: http://doi.org/10.1590/2175-8239-jbn-2018-0134.

30. Lopes LCC, Mota JF, Prestes J, Schincaglia RM, Silva DM, Queiroz NP, et al. Intradialytic resistance training improves functional capacity and lean mass gain in individuals on hemodialysis: a randomized pilot trial. Arch Phys Med Rehabil. 2019;100(11):2151-8. doi: http://doi.org/10.1016/j.apmr.2019.06.006. PubMed PMID: 31278924.

31. Valle F, Valle Pinheiro B, Almeida Barros AA, Ferreira Mendonça W, de Oliveira AC, de Oliveira Werneck G, et al. Effects of intradialytic resistance training on physical activity in daily life, muscle strength, physical capacity and quality of life in hemodialysis patients: a randomized clinical trial. Disabil Rehabil. 2020;42(25):3638-44. doi: http://doi.org/10.1080/09638288.2019.1606857. PubMed PMID: 31034264.

32. Dipp T, Macagnan FE, Schardong J, Fernandes RO, Lemos LC, Plentz RDM. Short period of high-intensity inspiratory muscle training improves inspiratory muscle strength in patients with chronic kidney disease on hemodialysis: a randomized controlled trial. Braz J Phys Ther. 2020;24(3):280-6. doi: http://doi.org/10.1016/j.bjpt.2019.04.003. PubMed PMID: 31122717.

33. Garcia RSA, Pinheiro BV, Lucinda LMF, Pimentel AL, Júnior JMP, Paula RB, et al. Association between exercise training in haemodialysis patients and burden of their family caregivers: A cross-sectional study. Nephrology (Carlton). 2020;25(4):332-8. doi: http://doi.org/10.1111/nep.13620. PubMed PMID: 31124254.

34. Cardoso RK, Araujo AM, Del Vechio FB, Bohlke M, Barcellos FC, Oses JP, et al. Intradialytic exercise with blood flow restriction is more effective than conventional exercise in improving walking endurance in hemodialysis patients: a randomized controlled trial. Clin Rehabil. 2020;34(1):91-8. doi: http://doi.org/10.1177/0269215519880235. PubMed PMID: 31603002.

35. Corrêa HL, Moura SRG, Neves RVP, Tzanno-Martins C, Souza MK, Haro AS, et al. Resistance training improves sleep quality, redox balance and inflammatory profile in maintenance hemodialysis patients: a randomized controlled trial. Sci Rep. 2020;10(1):11708. doi: http://doi.org/10.1038/s41598-020-68602-1. PubMed PMID: 32678132.

36. Exel AL, Lima PS, Urtado CB, Dibai-Filho AV, Vilanova CL, Sabino EFP, et al. Effectiveness of a resistance exercise program for lower limbs in chronic renal patients on hemodialysis: A randomized controlled trial. Hemodial Int. 2021;25(3):372-9. doi: http://doi.org/10.1111/hdi.12918. PubMed PMID: 33682262.

37. Neves RVP, Corrêa HL, Deus LA, Reis AL, Souza MK, Simões HG, et al. Dynamic not isometric training blunts osteo-renal disease and improves the sclerostin/FGF23/Klotho axis in maintenance hemodialysis patients: A randomized clinical trial. J Appl Physiol. 2021;130(2):508-16. doi: http://doi.org/10.1152/japplphysiol.00416.2020. PubMed PMID: 33242299.

38. Andrade FP, Borba GC, da Silva KC, Ferreira TS, de Oliveira SG, Antunes VVH, et al. Intradialytic periodized exercise improves cardiopulmonary fitness and respiratory function: A randomized controlled trial. Semin Dial. 2022;35(2):181-9. doi: http://doi.org/10.1111/sdi.13020. PMid:34536050.

39. Brito JS, Vargas D, da Silva GS, Marinho S, Borges NA, Cardozo LFMF, et al. Uremic toxins levels from the gut microbiota seem not to be altered by physical exercise in hemodialysis patients. Int Urol Nephrol. 2022;54(3):687-93. doi: http://doi.org/10.1007/s11255-021-02945-0. PubMed PMID: 34254218.

40. Paim F, Silveira KN, Thais Severo, Kupske V, Kupske JW, Krug MM, et al. Influência do treinamento resistido sobre variáveis de saúde de pacientes em hemodiálise. Lect Educ Física y Deport. 2022;26(284):66-81. doi: http://doi.org/10.46642/efd.v26i284.2475.

41. Pereira ABN, Santana LL, Rocha LDB, Cunha KDC, Rocha LSDO, Santos MCDS, et al. Physical exercise affects quality of life and cardiac autonomic modulation in patients with chronic kidney failure submitted to hemodialysis: a randomized clinical trial. Percept Mot Skills. 2022;129(3):696-713. doi: http://doi.org/10.1177/00315125221085811. PubMed PMID: 35426351.

42. Ribeiro HS, Cunha VA, Dourado GÍ, Duarte MP, Almeida LS, Baião VM, et al. Implementing a resistance training programme for patients on short daily haemodialysis: A feasibility study. J Ren Care. 2023;49(2):125-33. doi: http://doi.org/10.1111/jorc.12423. PubMed PMID: 35526118.

43. Teixeira MS, Ferrari F, Dipp T, Carvalho G, Bitencourt EDS, Saffi M, et al. Effects of intradialytic inspiratory muscle training at different intensities on diaphragm thickness and functional capacity: clinical trial protocol in patients undergoing haemodialysis. BMJ Open. 2023;13(1):e066778. doi: http://doi.org/10.1136/bmjopen-2022-066778. PubMed PMID: 36707111.

44. Barbalho-Moulim MC, Paro FM, Pedrosa DF, Serafim LM, Kuster E, Carmo WAD, et al. Effects of upper limbs' neuromuscular electrical stimulation (NMES) superimposed to voluntary contraction added to a protocol of intradialytic leg cycle ergometer exercise, in muscle strength, functional capacity and quality of life of adult patients with CKD: a randomized clinical trial protocol. Physiother Res Int. 2024;29(2):e2079. doi: http://doi.org/10.1002/pri.2079. PubMed PMID: 38477078.

45. Moraes IG, Brito CP, de Souza Francisco D, Faria LM, Luders C, de Brito CMM, et al. Efficacy of neuromuscular electrical stimulation with combined low and high frequencies on body composition, peripheral muscle function and exercise tolerance in patients with chronic kidney disease undergoing haemodialysis: a protocol for a randomised, double-blind clinical trial. BMJ Open. 2022;12(11):e062062. doi: http://doi.org/10.1136/bmjopen-2022-062062. PubMed PMID: 36351736.
