# Supplementary material for: Exercise prescriptions for patients on hemodialysis in Brazil: a scoping review
Source: J Bras Nefrol. 2024 Sep 20;46(4):e20240049. doi: 10.1590/2175-8239-JBN-2024-0049en (PMC11420935; doi:10.1590/2175-8239-JBN-2024-0049en)
Supplement: Supplementary file 3 [file 2175-8239-jbn-46-4-e20240049-supp04.pdf]

Braz. J. Nephrol. (J. Bras. Nefrol.)
https://doi.org/10.1590/2175-8239-JBN-2024-0049en

Supplementary Material to "Exercise prescriptions for patients on hemodialysis in Brazil: a scoping review"

Table S2. Characteristics of the exercise protocols.

Aerobic

Author	Year	Source	State	Setting	HD time	Intervention	Duration (weeks)	Weekly frequency (days)	Type	Duration (min)	Warm-up (min)	Intensity approach	Intensity tool	Intensity	Volume (min)	Cool-down (min)	Periodization	Supervision
Barbalho-Moulim et al.	2024	Article	ES	Intradialytic	First half	Cycle ergometer	8	3	continuous	30	5	subjective	Borg (0-10)	4-7	20	5	No progression/periodization	Not described
Belik et al.	2018	Article	SP	Intradialytic	First half	Cycle ergometer	17	3	continuous	30-45		objective	HRmax	65-75%	30-45		Progressively increased from 30- to 45-min	Not described
Brito et al.	2021	Article	RJ	Intradialytic	First half	Stationary bike	13	3	continuous	45	5	subjective and objective	Borg (0-10) and HR	Borg 4-5	35	5	No progression/periodization	Exercise physiologist
Cardoso et al.	2020	Article	RS	Intradialytic	Not described	Intradialytic cycling with blood flow restriction (50% in proximal thighs)	12	3	continuous	20		subjective and objective	HRmax and Borg scale (6-20)	60-76% and 10-13	20		Increase intensity in the last six weeks	Exercise physiologist
Ferreira et al.	2015	Article	PI	Intradialytic	First half	Cycle ergometer	12	3	continuous	60	5	objective	HRmax	60%	30	5	No progression/periodization	Not described

Author	Year	Source	State	Setting	HD time	Intervention	Duration (weeks)	Weekly frequency (days)	Type	Duration (min)	Warm-up (min)	Intensity approach	Intensity tool	Intensity	Volume (min)	Cool-down (min)	Periodization	Supervision
Guio et al.	2017	Article	RJ	Intradialytic	First half	Cycle ergometer	35	3	continuous	30		objective	reserve HR	50-80%			No progression/periodization	Research staff
Lima et al.	2013	Article	PR	Intradialytic	Not described	Cycle ergometer	8	3	continuous	40	10	objective	HRmax	60%	20	10	No progression/periodization	Not described
Morais et al.	2019	Article	SP	Intradialytic	First half	Cycle aerobic training	12	3	continuous	30		objective	HRmax	45-60%	30		No progression/periodization	Exercise physiologist
Reboredo et al.	2010	Article	AC	Intradialytic	First half	Cycle ergometer training	12	3	continuous	60	15	subjective	Borg (0-10)	4-6	35	3	No progression/periodization	Health professional
Silva et al.	2019	Article	MG	Intradialytic	First half	Cycloergometer training	17	3	continuous	30		objective	HRmax	65-75%	30		Progressively increase the load	Not described
Lima et al.	2013	Article	RS	Intradialytic	First half	Ergometric bicycle	8	3	continuous	20		subjective	Borg (0-10)	2-3	20		No progression/periodization	Not described
Pereira et al.	2022	Article	PA	Intradialytic	First half	Cycle ergometer	13	3	continuous	30	5	subjective and objective	HRmax and Borg (0-10)	70-80% and 3-4	20	5	Increase by 5% speed monthly	Health professional

HD, hemodialysis; RPE, rate of perceived exertion; Borg, RPE Borg scale; W, workload; VO_2, oxygen consumption.

Strength

Author(s)	Year	Source	State	Setting	HD time	Intervention	Duration (weeks)	Weekly frequency (days)	Duration (min)	Limbs	Warm-up	Intensity approach	Intensity tool	Intensity	Equipment	Sets	Repetitions	Rest interval (sec)	Cool-down (min)	Periodization	Supervision
Castro et al.	2018	Article	MG	Intradialytic	First half	Resistance training	39	3	50	lower and upper		subjective	RPE (6-20)	15-17	Free-weights	1-3	10-15	90-120		Increase volume and weight in 5%	Exercise physiologist
Correa et al.	2020	Article	SP	Intradialytic	First half	Resistance training	12	3	50	lower and upper		subjective	OMNI RPE	6-8	Free-weights and Elastic-bands	3	8-12	120		No progression/periodization	Exercise physiologist
Exel et al.	2021	Article	AL	Intradialytic	First half	Resistance exercise	8	3	30	lower		objective	Isometric maximal strength of quadriceps	50%	Free-weights	3	10	120		Increase 0.5 kg in the 6th, 12th, and 18th sessions	Not described
Lopes et al.	2019	Article	PR	Intradialytic	Second half	Resistance training	12	3	20-40	lower	yes	subjective	OMNI-RES		Free-weights and Elastic-bands	1-4	8-20	60		Increase intensity and sets over the weeks	Health professional
Lopes et al.	2019	Article	GO	Intradialytic	Second half	Resistance training	12	3	20-40	lower	yes	subjective	OMNI-RES		Free-weights and Elastic-bands	1-3	15-20	60		Increase sets over the weeks	Health professional
Martins et al.	2010	Article	GO	Pre-dialysis		Resistance exercises	13	2	20-30											No progression/periodization	Health professional
Moraes et al.	2013	Article	SP	Intradialytic	First half	Resistance exercise training	26	3		lower		objective	1RM	60-70%	Free-weights and Elastic-bands			60		1RM retested every 12 sessions	Physiotherapist; Exercise physiologist
Neves et al.	2021	Article	RJ	Pre-dialysis		Resistance exercises	26	3	40	lower and upper		subjective	OMNI-RES	6-8	Free-weights, Elastic-bands, and Bodyweight			60		No progression/periodization	Not described
Neves et al.	2021	Article	SP	Pre-dialysis		Isometric resistance exercises	26	3	40	lower and upper		subjective	OMNI-RES	6-8	Free-weights, Elastic-bands, and Bodyweight			60		No progression/periodization	Not described

Author(s)	Year	Source	State	Setting	HD time	Intervention	Duration (weeks)	Weekly frequency (days)	Duration (min)	Limbs	Warm-up	Intensity approach	Intensity tool	Intensity	Equipment	Sets	Repetitions	Rest interval (sec)	Cool-down (min)	Periodization	Supervision
Orcy et al.	2012	Article	SP	Intradialytic	First half	Resistance exercises	10	3	30	lower and upper		subjective			Free-weights and Elastic-bands	2	10-15			Weights adjusted when 15 reps were done	Physiotherapist
Pellizzaro et al.	2013	Article	RS	Intradialytic	First half	Knee extensor muscles training	10	3		lower		objective	1RM	50%	Free-weights	3	15	60		Intensity reassessed at 30-day	Not described
Ribeiro et al.	2013	Article	RS	Intradialytic	Not described	Resistance training	8	3		lower and upper	yes	objective	Manual strength			3	12			Increase intensity in 10% after every 6 sessions	Not described
Rocha et al.	2010	Article	SP	Intradialytic	First half	Resistance exercise training	9	3	25	lower and upper					Free-weights	3	10	15		No progression/periodization	Physiotherapist
Rosa et al.	2018	Article	MG	Intradialytic and Pre-dialysis	Not described	Progressive resistance training	12	3	40-50	lower and upper		objective	RM zone		Free-weights and Elastic-bands	2	15-20		yes	Adjust RM zone	Exercise physiologist
Valle et al.	2019	Article	SP	Intradialytic	First half	Resistance training	12	3		lower and upper		subjective	Borg (0-10)	3-5	Free-weights	2-3	10			Increase volume at the 3rd week	Health professional
Lima et al.	2013	Article	MG	Intradialytic	First half	Strength exercises	8	3		lower		objective	1RM	40	Free-weights	3	15			1RM reassessed every 15 days	Not described
Paim et al.	2022	Article	RS	Intradialytic	First half	Resistance training	13	3	20-45	lower and upper	stretching	subjective	Borg (0-10)	6-8	Free-weights and elastic-bands	3	10 or 30”	60	stretching	No progression/periodization	Physiotherapist; Exercise physiologist
Ribeiro et al.	2022	Article	DF	Intradialytic	First and/or second half	Resistance training	34	2	25-35	lower and upper	stretching	subjective	Borg (0-10)	3-7	Free-weights and elastic-bands	1-3	11-15	60	yes	Systematic increase in intensity every 12 weeks	Physiotherapist; Exercise physiologist

AVF, arteriovenous fistula; HD, hemodialysis; RPE, rate of perceived exertion; Borg, RPE Borg scale; OMNI-RES, OMNI resistance exercise scale; RM, repetition maximum.

Combined (aerobic)

Author(s)	Year	Source	State	Setting	HD time	Intervention	Session	Order	Duration (weeks)	Weekly frequency (days)	Type	Duration (min)	Warm-up (min)	Intensity approach	Intensity tool	Intensity	Volume (min)	Cool-down (min)	Periodization	Supervision
Andrade et al.	2021	Article	RS	Intradialytic	First half	Bicycle and lower limb resistance exercises	Same session	Aerobic - strength	12	3	continuous	30	5	subjective	Speed (km/h)	Corresponding speed to a 6 RPE	20	5	Increase speed in 3km/h and 1RM% at 5- and 9-week	Physiotherapist
Garcia et al.	2020	Article	MG	Intradialytic	First half	Resistance and aerobic exercises	Same session	Strength - aerobic		2		35		subjective	Borg (0-10)	4-6	35		No progression/periodization	Physiotherapist
Marchesan et al.	2013	Article	RS	Intradialytic		Stationary bicycle and resistance exercises	Same session	Aerobic - strength	17	3	continuous	13-48	3	subjective and objective	Borg (0-10) and HRmax	3-4 and 60-70%	10-45		Increase intensity and volume throught sessions	Exercise physiologist
Orcy et al.	2012	Article	RS	Intradialytic	First half	Combined aerobic and resistance exercises	Same session	Aerobic - strength	10	3	continuous	20		subjective	Borg (6-20)	13-14	20		Weights adjusted when 15 reps were done	Physiotherapist
Silva et al.	2013	Article	MG	Intradialytic	First half	Combined cycling and resistance exercises	Same session	Aerobic - strength	70	3	continuous	10	2	objective	HR	60-70%	6	2	No progression/periodization	Physiotherapist
Tomich et al.	2014	Article	PA	Interdialytic		Treadmill or bike and muscle strengthening exercises	Same session	Aerobic - strength	6	3	continuous	35	5	subjective and objective	HRmax and Borg (6-20)	50-70% and 13	30		No progression/periodization	Physiotherapist

HD, hemodialysis; RPE, rate of perceived exertion; Borg, RPE Borg scale; W, workload; VO_2, oxygen consumption.

Combined (strength)

Author(s)	Year	Source	State	Setting	HD time	Intervention	Session	Order	Duration (weeks)	Weekly frequency (days)	Duration (min)	Limbs	Warm-up	Intensity approach	Intensity tool	Intensity	Equipment	Sets	Repetitions	Rest interval (sec)	Cool-down (min)	Periodization	Supervision
Andrade et al.	2021	Article	RS	Intradialytic	First half	Bicycle and lower limb resistance exercises	Same session	Aerobic - strength	12	3		lower		objective	1RM	10-15%	free-weights	2	12-18	60		Increase speed in 3km/h and 1RM% at 5- and 9-week	Physiotherapist
Garcia et al.	2020	Article	MG	Intradialytic	First half	Resistance and aerobic exercises	Same session	Strength - aerobic		2		lower and upper	yes	subjective	Borg (0-10)	4-6	free-weights	3	15			No progression/periodization	Physiotherapist
Marchesan et al.	2013	Article	RS	Intradialytic		Stationary bicycle and resistance exercises	Same session	Aerobic - strength	17	3		lower and upper						3	12-15			Increase intensity and volume throught sessions	Exercise physiologist
Orcy et al.	2012	Article	RS	Intradialytic	First half	Combined aerobic and resistance exercises	Same session	Aerobic - strength	10	3	10	lower and upper		subjective			free-weights and elastic bands	2	10-15			Weights adjusted when 15 reps were done	Physiotherapist
Silva et al.	2013	Article	MG	Intradialytic	First half	Combined cycling and resistance exercises	Same session	Aerobic - strength	70	3	10	lower and upper					free-weights and elastic bands					No progression/periodization	Physiotherapist
Tomich et al.	2014	Article	PA	Interdialytic		Treadmill or bike and muscle strengthening exercises	Same session	Aerobic - strength	6	3	15-20	lower					free-weights and elastic bands	1-4	10-15		Strecthing 5min	No progression/periodization	Physiotherapist

AVF, arteriovenous fistula; HD, hemodialysis; RPE, rate of perceived exertion; Borg, RPE Borg scale; OMNI-RES, OMNI resistance exercise scale; RM, repetition maximum.

Respiratory

Author(s)	Year	Source	State	Setting	HD time	Intervention	Duration (weeks)	Weekly frequency (days)	Duration (min)	Warm-up	Intensity approach	Intensity tool	Intensity	Volume	Sets	Repetitions	Rest interval (sec)	Cool-down	Periodization	Supervision
Campos et al.	2018	Article	CE	Intradialytic	First half	Intradialytic respiratory training	8	3	30-40		objective	Resistance (cmH20)	15-20	30-40					Increase volume and intensity in the last 12 sessions	Physiotherapist
Dipp et al.	2020	Article	RS	Intradialytic and home-based		Inspiratory muscle training	5	6			objective	Maximal inspiratory pressure	50-70%		5	10	120		Increase intensity weekly	Physiotherapist
Figueiredo et al.	2018	Article	MG	Intradialytic	First half	Inspiratory muscle training	8	3			objective	Maximal inspiratory pressure	50%	15 inspirations	3	15	60		Reevaluate MIP every six sessions	Not described
Medeiros et al.	2019	Article	PE	Interdialytic		POWERbreathe inspiratory muscle training	8	7			objective	Maximal inspiratory pressure	50%	Twice a day	3	30 breaths	60		Inspiratory muscle strength weekly reassessed	Physiotherapist
Pellizzaro et al.	2013	Article	RS	Intradialytic	First half	Inspiratory muscle training	10	3			objective	Maximal inspiratory pressure	50%		3	15 inspirations	60		Intensity reassessed at 30-day	Not described
Silva et al.	2011	Article	SP	Intradialytic		Inspiratory muscle training	8	3	15-30		objective	Maximal inspiratory pressure	40%			5	60		Increase volume after 4 weeks	Not described
Teixeira et al.	2023	Article	RS	Intradialytic	First half	POWERbreathe Inspiratory muscle training	12	3			objective	Maximal inspiratory pressure	30%		3	15	60		Reassessments of MIP every 15 days	Not described
Teixeira et al.	2023	Article	RS	Intradialytic	First half	POWERbreathe Inspiratory muscle training	12	3			objective	Maximal inspiratory pressure	50%		3	15	60		Reassessments of MIP every 15 days	Not described

HD, hemodialysis; MIP, muscular inspiratory pressure

Mobility

Author(s)	Year	Source	State	Setting	HD time	Intervention	Duration (weeks)	Weekly frequency (days)	Duration (min)	Warm-up	Intensity approach	Intensity tool	Intensity	Volume	Sets	Repetitions	Rest interval (sec)	Cool-down	Periodization	Supervision
Padulla et al.	2011	Article	SP			Kinesiotherapy with mobility exercises	13	3	45					Mobility and breathing exercises					No progression/periodization	Physiotherapist
Sanchez et al.	2018	Article	GO	Intradialytic	First half	Breathing, mobility, and stretching exercises	8	3	25 min					Stretching for 15 seconds; mobility with five repetitions; and respiratory movements	3	5			No progression/periodization	Physiotherapist
Soares et al.	2011	Article	PR	Intradialytic	First half	Mobility, stretching, and relaxation exercises	13	2	25-30										No progression/periodization	Physiotherapist

HD, hemodialysis

Virtual Reality

Author(s)	Year	Source	State	Setting	HD time	Intervention	Duration (weeks)	Weekly frequency (days)	Duration (min)	Warm-up	Intensity approach	Intensity tool	Intensity	Volume	Sets	Repetitions	Rest interval (sec)	Cool-down	Periodization	Supervision
Maynard et al.	2019	Article	SE	Intradialytic	First half	Virtual Reality - Wii -, cycle ergometer, and elastic band exercises	12	3			subjective	Borg (6-20)	12-14	Nine motor demands					Changes in the type of games and exercises every 2 weeks	Physiotherapy

HD, hemodialysis; RPE, rate of perceived exertion.

Neuroelectric stimulation

Author(s)	Year	Source	State	Setting	HD time	Intervention	Duration (weeks)	Weekly frequency (days)	Duration (min)	Warm-up	Intensity approach	Intensity tool	Intensity	Volume	Sets	Repetitions	Rest interval	Cool-down	Periodization	Supervision
Barbalho-Moulim et al.	2024	Article	ES	Intradialytic	First half	Unilateral NMES on the upper limb without arteriovenous fistula	8	3	20	30 min of aerobic cycle ergometer	objective		80 Hz; 350 µs			5 seconds	10 seconds		The intensity will be individually adjusted progressively to achieve the greatest pain-free forceful contraction	Not described
Moraes et al.	2022	Article	SP	Intradialytic	Second third	NMES of vastus lateralis and medialis	8	3	60	4 min of 20-80% of intensity	objective		20-70 Hz; 400 µs	30 min with 20 Hz, followed by 30 min of 70 Hz		10 seconds	20 seconds	4 min of 80-20% of intensity	Intensity will be increased according to the patient tolerance	Physical therapist
Moraes et al.	2022	Article	SP	Intradialytic	Second third	NMES of vastus lateralis and medialis	8	3	60	5 min of 20-80% of intensity	objective		20 Hz; 400 µs	60 min		10 seconds	20 seconds	5 min of 80-20% of intensity	Intensity will be increased according to the patient tolerance	Physical therapist
Moraes et al.	2022	Article	SP	Intradialytic	Second third	NMES of vastus lateralis and medialis	8	3	60	5 min of 20-80% of intensity	objective		70 Hz; 400 µs	60 min		10 seconds	20 seconds	5 min of 80-20% of intensity	Intensity will be increased according to the patient tolerance	Physical therapist
Roxo et al.	2016	Article	SP	Intradialytic	Not described	NMES	8	3	30		subjective and objective	According to the tolerance	50 Hz	30 min		2 seconds	10 seconds		Not described	Not described

HD, hemodialysis; Hz, hertz; mA, milliampere; NMES, neuromuscular electrical stimulation.

Vibration platform

Author(s)	Year	Source	State	Setting	Intervention	Duration (weeks)	Weekly frequency (days)	Duration (min)	Warm-up	Intensity approach	Intensity tool	Intensity	Volume	Sets	Repetitions	Rest interval (sec)	Cool-down	Periodization	Supervision
Fuzari et al.	2018	Article	PE	Interdialytic	Whole body vibration	12	2	10-20min		objective	Hertz	35	Semi-squat static position		60sec	30		Increase duration during weeks	Not described
