# Supplementary material for: Exercise prescriptions for patients on hemodialysis in Brazil: a scoping review
Source: J Bras Nefrol. 2024 Sep 20;46(4):e20240049. doi: 10.1590/2175-8239-JBN-2024-0049en (PMC11420935; doi:10.1590/2175-8239-JBN-2024-0049en)
Supplement: Translation

# Translation: Prescrições de exercícios físicos para pacientes em hemodiálise no Brasil: uma revisão de escopo

**DOI:** 10.1590/2175-8239-JBN-2024-0049pt

**Authors:** Heitor S. Ribeiro, Francini P. Andrade, Diogo V. Leal, Juliana S. Oliveira, Kenneth R. Wilund, Maycon M. Reboredo, João L. Viana

**Affiliations:** 1 Universidade da Maia, Centro de Investigação em Ciências do Desporto, Ciências da Saúde e Desenvolvimento Humano, Maia, Portugal. Universidade da Maia, Centro de Investigação em Ciências do Desporto Ciências da Saúde e Desenvolvimento Humano Maia Portugal; 2 Universidade de Brasília, Faculdade de Medicina, Brasília, DF, Brasil. Universidade de Brasília Faculdade de Medicina Brasília DF Brasil; 3 Universidade Federal do Rio Grande do Sul, Programa de Pós-Graduação em Ciências Pneumológicas, Porto Alegre, RS, Brasil. Universidade Federal do Rio Grande do Sul Programa de Pós-Graduação em Ciências Pneumológicas Porto Alegre RS Brasil; 4 The University of Sydney and Sydney Local Health District, Institute for Musculoskeletal Health, Sidney, Austrália. The University of Sydney and Sydney Local Health District Institute for Musculoskeletal Health Sidney Austrália; 5 University of Illinois Urbana-Champaign, Department of Kinesiology and Community Health, Urbana, Estados Unidos. University of Illinois Urbana-Champaign Department of Kinesiology and Community Health Urbana Estados Unidos; 6 Universidade Federal de Juiz de Fora, Faculdade de Medicina, Juiz de Fora, MG, Brasil. Universidade Federal de Juiz de Fora Faculdade de Medicina Juiz de Fora MG Brasil

**Keywords:** Insuficiência Renal Crônica, Diálise, Exercício Físico, Treinamento Resistido, Treino Aeróbico

## Abstract

**Introdução:**

O exercício físico está sendo incorporado ao tratamento de pacientes em hemodiálise, porém pouco se sabe sobre as principais características dessas intervenções.

**Objetivo:**

Descrever os protocolos de exercício físico prescritos para pacientes em hemodiálise no Brasil.

**Métodos:**

Uma revisão de escopo foi conduzida de acordo com as diretrizes JBI e Prisma-ScR. Foram realizadas pesquisas na Medline, Embase e em outras três bases de dados até maio de 2024. Outras fontes (sites, livros e diretrizes) também foram pesquisadas. Foram incluídas evidências de pacientes em hemodiálise, descrevendo protocolos de exercício físico em todos os ambientes e desenhos no Brasil.

**Resultados:**

Encontradas 45 evidências, resultando em 54 protocolos de exercício físico de 16 estados brasileiros. O exercício de força (33,3%), seguido do exercício aeróbico (22,2%), foi o mais prescrito para ser realizado durante a diálise (85,2%). Os profissionais mais prevalentes na supervisão dos programas foram fisioterapeutas e profissionais de educação física (37,0% e 18,5%, respectivamente). Todos os protocolos adotaram os princípios de treinamento tipo e frequência, enquanto a progressão foi adotada em apenas 53,7%. A frequência mais prescrita foi três vezes por semana (88,9%). A intensidade do exercício foi determinada predominantemente por métodos subjetivos (33,3%).

**Conclusão:**

Os exercícios aeróbicos e de força durante a diálise foram as modalidades mais prescritas no Brasil, com a maioria dos programas sendo adequadamente supervisionada por profissionais qualificados. No entanto, os protocolos existentes não adotaram a progressão sistemática no decorrer da intervenção, o que seria adequado para proporcionar melhores respostas e adaptações fisiológicas.

## Introdução

A hemodiálise é a terapia renal substitutiva mais prescrita no Brasil. Dados brasileiros do inquérito sobre diálise de 2022 mostraram que 95% dos pacientes com falência renal faziam esse tratamento^1^. Pacientes em hemodiálise comumente apresentam comportamento sedentário^2^ e incapacidade física^3–6^, o que aumenta sua morbidade e mortalidade. Intervenções com exercícios físicos têm sido introduzidas como uma tentativa de modificar tal cenário^7^, mas ainda falta sua implementação como parte da rotina de tratamento^8^. Além disso, há a necessidade de evidências que descrevam protocolos de exercícios em detalhes para apoiar a expansão de programas viáveis.

Anteriormente, os resultados da análise mundial de uma revisão de escopo mostraram que o Brasil foi o principal país a relatar protocolos de exercícios físicos para essa população^9^. Contudo, Barros et al.^10^ descobriram recentemente que apenas 16% dos centros de diálise no Brasil tinham um programa de exercício intradialítico integrado à sua rotina clínica. Na tentativa de aumentar a conscientização e o conhecimento da Sociedade Brasileira de Nefrologia para ajudar na implementação de programas de exercícios físicos, realizamos uma análise secundária de uma revisão de escopo para descrever as prescrições de exercícios físicos para pacientes em hemodiálise no Brasil.

## Métodos

O protocolo da nossa revisão de escopo e o artigo com os dados mundiais foram publicados anteriormente^9,11^ e mais detalhes da metodologia podem ser vistos nessas publicações. Brevemente, foram adotadas as diretrizes JBI^12^ e Prisma-ScR^13^ de revisão de escopo. Foi realizada uma estratégia de busca completa nas bases de dados Medline, Embase, SportDiscus, Cinahl e Lilacs utilizando termos relacionados a “hemodiálise”, “diálise”, “exercício físico” e “treinamento físico”. A estratégia de busca completa para cada base de dados pode ser vista no Supplementary Material 1. As bases de dados foram pesquisadas desde o início até dezembro de 2021 e a busca manual adicional por novas evidências brasileiras foi feita até maio de 2024.

Foi seguida a estrutura do PCC e escolhida qualquer evidência de relatórios/estudos com pacientes adultos em hemodiálise (participantes), prescrevendo intervenções com exercícios físicos (conceito), em todos os ambientes e desenhos de estudo no Brasil (contexto). Dois revisores independentes (HR e FA) selecionaram títulos e resumos. A leitura do texto completo foi feita pelo revisor principal (HR). As divergências foram discutidas com um revisor adicional (DL).

A extração dos dados relevantes foi realizada pelo revisor principal (HR) e dupla verificação por outros (FA e DL) em uma planilha adaptada^14^. Os dados relevantes incluíram detalhes sobre a prescrição do exercício (por exemplo, tipo, frequência, duração, local, volume, progressão, intensidade, profissionais envolvidos, periodização etc.). As divergências foram resolvidas com um revisor adicional (JV).

Os dados foram descritos de acordo com a diretriz Prisma-ScR^13^. Foram realizadas análises dos protocolos de acordo com os princípios de exercício do American College of Sports Medicine (ACSM) (FITT-VP: frequência, intensidade, tempo, tipo, volume e progressão)^15^. Uma descrição mais detalhada da análise dos dados pode ser vista em outra publicação^9^.

## Resultados

### Seleção de Estudos

Foram encontrados 21.312 registros; destes, 285 foram incluídos em nossa principal revisão mundial^8^. Do Brasil, 39 foram incluídos nessa análise secundária. Em uma busca adicional conduzida em maio de 2024, foram encontrados 6 artigos publicados entre 2022 e 2024, rendendo 54 protocolos de exercícios (Figura 1; lista completa dos artigos no Supplementary Material 2). Todos os protocolos de exercícios foram relatados em artigos originais.

**Figura 1:** Fluxograma da revisão de escopo.

### Características

Os protocolos incluídos vieram de 16 estados e de todas as regiões brasileiras. A Figura 2 mostra que o estado mais prevalente foi São Paulo (n = 14; 25,9%). O treinamento de força (n = 18; 33,3%) foi o tipo mais prescrito. A Table S2 mostra as características dos protocolos incluídos.

**Figura 2:** Mapa de calor geográfico dos protocolos de exercícios físicos prescritos para pacientes em hemodiálise no Brasil.

### Constatações Gerais

A Tabela 1 descreve as principais características dos programas de exercícios. A duração mediana das intervenções foi de 12 [intervalo interquartil: 8 – 13] semanas. O exercício foi prescrito principalmente durante a diálise (ou seja, intradialítica; n = 45; 83,3%). Para os programas de exercícios que prescreveram qualquer componente intradialítico, a intervenção foi realizada principalmente durante a primeira metade da sessão de diálise (n = 35; 77,8%). Os profissionais mais prevalentes na supervisão dos programas de exercícios foram fisioterapeutas e profissionais de educação física (n = 20; 37,0% e n = 10; 18,5%, respectivamente).

**Tabela 1:** Características dos protocolos de exercício físico

	Aeróbico (n = 12)	Força (n = 18)	Combinado* (n = 6)		Mobilidade (n = 3)	Respiratório (n = 8)	Estimulação elétrica neuromuscular (n = 5)	Realidade virtual (n = 1)	Plataforma vibratória (n = 1)
Duração (semanas), mediana [IIQ]	12 [12 – 17]	12 [10 – 26]	12 [8 – 44]		13 [8 – 13]	8 [8 – 12]	8	12	12
**Ambiente**, n (%)								
Intradialítico^#^	12 (100)	15 (83,3)	5 (83,3)		2 (66,7)	6 (75,0)	5 (100)	1 (100)	0
Interdialítico	0	0	1 (16,7)		0	1 (12,5)	0	0	1 (100)
Pré-hemodiálise	0	3 (16,7)	0		0	0	0	0	0
Pós-hemodiálise	0	0	0		0	0	0	0	0
Pré e intradialítico	0	0	0		0	0	0	0	0
Domiciliar	0	0	0		0	0	0	0	0
Domiciliar e intradialítico	0	0	0		0	1 (12,5)			
Não reportado	0	0	0		1 (33,3)	0	0	0	0
**Momento da diálise**^†^, n (%)								
Primeira metade	10 (83,3)	11 (73,3)	4 (80,0)		2 (100)	5 (62,5)	4 (80,0)	1 (100)	–
Segunda metade	0	3 (20,0)	0		0	0	0	0	–
Não reportado	2 (16,7)	2 (13,3)	1 (20)		0	1 (12,5)	1 (20,0)	0	–
**Supervisão profissional**^†^, n (%)								
Fisioterapeuta	0	5 (27,8)	5 (83,3)		3 (100)	3 (37,5)	3 (60,0)	1 (100)	0
Profissional de educação física	3 (25,0)	6 (33,3)	1 (16,7)		0	0	0	0	0
Profissional de saúde	2 (16,7)	4 (22,2)	0		0	0	0	0	0
Não supervisionado	0	0	0		0	1 (12,5)	0	0	0
Não reportado	8 (66,7)	8 (44,4)	0		0	5 (62,5)	2 (40,0)	0	1 (100)
**Componentes da sessão**, n (%)			**Aeróbico**	**Força**					
Aquecimento	6 (50,0)	5 (27,8)	4 (66,7)	1 (16,7)	0	0	4 (80,0)	0	0
Condicionamento	11 (91,7)	14 (77,8)	6 (100)	5 (83,3)	2 (66,7)	8 (100)	5 (100)	1 (100)	1 (100)
Volta à calma	6 (50,0)	3 (16,7)	2 (33,3)	1 (16,7)	0	0	3 (60,0)	0	0

Abreviação: IIQ, intervalo interquartil.

Notas: *Combinação de treinamento aeróbico e de força.

^#^Apenas durante a diálise, não inclui outras configurações.

^†^A soma pode ser maior que 100% porque alguns programas de exercícios incluíam mais de um.

Existem valores faltantes devido à ausência de dados em alguns estudos incluídos.

Os princípios de exercício físico adotados nos protocolos de exercícios prescritos estão demonstrados na Figura 3. Todos os protocolos adotaram tipo e frequência, mas a progressão foi adotada em apenas 53,7% (n = 29). A maioria dos protocolos prescrevia exercícios três dias por semana (n = 47; 88,9%) e utilizava métodos subjetivos (n = 18, 33,3%) para determinar a intensidade do exercício, enquanto 14 (25,9%) protocolos não relatavam controle de intensidade.

**Figura 3:** Princípios de treinamento FITT-VP (ACSM) adotados nos protocolos mais prescritos.

Abreviações: ACSM, American College of Sports Medicine; FITT-VP, Frequência, Intensidade, Tempo, Tipo, Volume e Progressão.

### Características dos Protocolos se Exercícios

#### Aeróbico

O cicloergômetro foi utilizado em todos os protocolos aeróbicos (n = 12, 100%) e um incluiu restrição parcial do fluxo sanguíneo nos membros inferiores. Todos os protocolos prescreveram exercícios três dias por semana, e a maioria monitorou a intensidade do exercício usando métodos objetivos (n = 9; 75,0%), principalmente por meio de escalas de frequência cardíaca (n = 9; 75,0%) e taxa de percepção subjetiva de esforço (PSE) (n = 6; 50,0%).

#### Força

Seis protocolos de força incluíram exercícios para membros inferiores (33,3%) e 11 combinados para membros inferiores e superiores (61,1%). Seis protocolos utilizaram halteres e pesos livres (33,3%). Dois estudos incorporaram exercícios com peso corporal (11,1%). A maioria dos protocolos prescrevia exercícios três dias por semana (n = 16, 88,9%) e monitorava a intensidade do exercício utilizando métodos subjetivos por meio de escalas de PSE (n = 10, 55,6%).

#### Combinado

Todos os protocolos combinados prescreveram treinamento aeróbico e de força na mesma sessão (n = 6, 100%), sendo o aeróbio realizado predominantemente primeiro (n = 5, 83,3%).

#### Componente Aeróbico

O ciclismo foi prescrito para todos os protocolos, e um também incluiu caminhada/corrida em esteira^16^. Cinco protocolos prescreveram exercícios três dias por semana (83,3%), e a maioria monitorou a intensidade do exercício usando métodos subjetivos (n = 5, 83,3%), principalmente por meio de escalas de PSE (n = 4, 66,7%).

#### Componente de Força

Quatro protocolos incluíram exercícios para membros inferiores e superiores (66,6%) e dois apenas para membros inferiores (33,3%). A maioria dos protocolos utilizou halteres e pesos livres (n = 5; 83,3%) e um incorporou faixas elásticas ou bolas (16,7%). Cinco protocolos prescreviam exercícios três dias por semana (83,3%) e apenas três descreviam monitoramento da intensidade do exercício (50%).

#### Respiratório

As intervenções respiratórias (n = 8) foram realizadas principalmente três dias por semana (n = 6; 75,0%) e métodos objetivos de intensidade aplicados em todas, principalmente através da pressão inspiratória máxima (n = 7; 87,5%), variando de 40 a 70%.

#### Mobilidade

Os protocolos de mobilidade (n = 3) foram prescritos principalmente três dias por semana (n = 2, 66,3%), com duração variando de 25 a 45 minutos. Nenhuma abordagem de intensidade ou progressão foi descrita.

#### Estimulação Elétrica Neuromuscular

Cinco protocolos utilizaram estimulação elétrica neuromuscular três dias por semana (100%), com sessões que duraram de 20 a 60 minutos cada. A intensidade foi objetivamente prescrita através da frequência de pulso (100%), que variou de 20 a 80 Hz.

#### Realidade Virtual

Um único protocolo utilizou *exergames* não imersivos e foi prescrito três dias por semana. A intensidade do exercício foi monitorada através da PSE, variando de 12 a 14 na escala de Borg de 6 a 20, representando uma intensidade um pouco difícil a difícil.

#### Plataforma Vibratória

Um protocolo de exercício utilizou plataformas vibratórias na frequência de 35 Hz. O protocolo consistia em realizar semiagachamentos isométricos consecutivos de 30 segundos separados por períodos de descanso de 30 segundos por um total de 10 a 20 minutos, duas vezes por semana.

## Discussão

Nossa revisão de escopo descreveu os protocolos de exercícios físicos prescritos para pacientes em hemodiálise no Brasil. As evidências vieram principalmente dos estados de São Paulo e Rio Grande do Sul, sendo o treinamento de força o tipo de exercício físico mais prescrito. Os fisioterapeutas supervisionaram a maior parte dos protocolos. O princípio da progressão sistemática foi aplicado em apenas metade dos protocolos, enquanto a intensidade do exercício foi predominantemente determinada por métodos subjetivos. A frequência de exercício foi principalmente três vezes por semana e durante a diálise, o que mostra a necessidade de uma abordagem mais holística ao exercício e à atividade física nessa população, incluindo um estilo de vida que considera “mover-se mais”^17^.

Diferentemente das análises mundiais realizadas, os exercícios de força, mas não o aeróbico, foi o tipo mais prescrito entre os protocolos incluídos^9^. O exercício aeróbico geralmente requer supervisão profissional mínima e menor custo. No Brasil, o exercício físico só pode ser prescrito e supervisionado por fisioterapeutas e/ou profissionais de educação física, o que leva a acreditar que os exercícios de força têm sido mais amplamente adotados do que nos demais países do mundo, bem como por serem mais baratos e exigirem equipamentos mais simples quando comparados aos exercícios aeróbicos por cicloergômetro. Porém, mesmo com esse acompanhamento profissional, outros tipos de programas de exercícios (por exemplo, estimulação elétrica neuromuscular, realidade virtual e plataforma vibratória) têm sido pouco prescritos. Tais intervenções são geralmente caras e requerem equipamentos modernos, tornando mais difícil implementá-las na rotina clínica.

Em nossa análise global^9^, um grande número de protocolos não descreveu adequadamente as variáveis relacionadas ao exercício físico recomendadas pelo ACSM (ou seja, princípios FITT-VP)^15^, entretanto, nesta análise brasileira houve maior quantidade de protocolos de exercícios que seguiram todos ou a maioria dos princípios. Contudo, a progressão foi pouco adotada nos programas aeróbicos e combinados. A progressão adequada do exercício desempenha um papel importante na produção de adaptações fisiológicas em longo prazo e pode ter impacto nos benefícios para a saúde^18,19^. Portanto, são necessários esforços das sociedades brasileiras de nefrologia, fisioterapia e educação física para fornecer diretrizes sobre a implementação, a prescrição, o monitoramento e a supervisão de exercícios para pacientes em hemodiálise, como feito recentemente pela UK Renal Association^20^. O recém-criado Grupo Brasileiro de Reabilitação em Nefrologia (GBREN)^21,22^ funciona até agora como uma rede colaborativa para apoiar profissionais do exercício que têm trabalhado nessa área. Na prática clínica, até que haja uma diretriz nacional, recomendamos que os profissionais envolvidos na prescrição e supervisão de exercícios físicos nessa população sigam recomendações como as da ACSM^15^.

Até onde sabemos, esta é a primeira revisão de escopo que descreve as evidências sobre como os exercícios físicos têm sido prescritos para pacientes em hemodiálise no Brasil. Seguimos diretrizes bem reconhecidas, conduzimos uma estratégia de pesquisa abrangente e incluímos a maioria dos tipos de exercícios, o que contribuiu para um número robusto de estudos incluídos. Apesar desses pontos fortes, existem algumas limitações. A leitura do texto completo foi feita apenas pelo revisor principal, mas, para minimizar esse viés, a extração dos dados foi verificada novamente por outros dois. Além disso, a estratégia de busca não incluiu termos brasileiros específicos nem o idioma português brasileiro, o que pode ter impactado a captura de publicações que não sejam em inglês.

Em conclusão, as intervenções com exercícios de força e aeróbicos foram as mais prescritas no Brasil, porém outras modalidades estão sendo recentemente prescritas. Contudo, encontramos um baixo número de protocolos que adotaram progressão sistemática dos princípios do treinamento físico no decorrer da intervenção. Protocolos futuros deverão adotar esses critérios para proporcionar melhores respostas e adaptações fisiológicas. Portanto, acreditamos que nossas descobertas podem fornecer à comunidade da nefrologia melhor compreensão de como fornecer, implementar, monitorar e supervisionar programas de exercício físico para pessoas em hemodiálise. Além disso, as políticas públicas devem incentivar o aumento da presença de profissionais qualificados para supervisionar os programas de exercício físico (sabidamente, fisioterapeutas e profissionais de educação física).

## Material Suplementar

O seguinte material on-line está disponível para o presente artigo:

Supplementary Material 1 – Search strategies.

Supplementary Material 2 – References of the included reports.

Table S1 – Preferred Reporting Items for Systematic reviews and Meta-Analyses extension for Scoping Reviews (Prisma-ScR) Checklist.

Table S2 – Characteristics of the exercise protocols.
